# Abrupt changes in the patterns and complexity of anterior cingulate cortex activity when food is introduced into an environment

**DOI:** 10.3389/fnins.2013.00074

**Published:** 2013-05-23

**Authors:** Barak F. Caracheo, Eldon Emberly, Shirin Hadizadeh, James M. Hyman, Jeremy K. Seamans

**Affiliations:** ^1^Department of Psychiatry, Brain Research Centre, University of British ColumbiaVancouver, BC, Canada; ^2^Department of Physics, Simon Fraser UniversityBurnaby, BC, Canada

**Keywords:** prefrontal cortex, entropy, foraging, Markov, HMM, Kolmogorov complexity

## Abstract

Foraging typically involves two distinct phases, an exploration phase where an organism explores its local environment in search of needed resources and an exploitation phase where a discovered resource is consumed. The behavior and cognitive requirements of exploration and exploitation are quite different and yet organisms can quickly and efficiently switch between them many times during a foraging bout. The present study investigated neural activity state dynamics in the anterior cingulate sub-region of the rat medial prefrontal cortex (mPFC) when a reliable food source was introduced into an environment. Distinct and largely independent states were detected using a Hidden Markov Model (HMM) when food was present or absent in the environment. Measures of neural entropy or complexity decreased when rats went from exploring the environment to exploiting a reliable food source. Exploration in the absence of food was associated with many weak activity states, while bouts of food consumption were characterized by fewer stronger states. Widespread activity state changes in the mPFC may help to inform foraging decisions and focus behavior on what is currently most prominent or valuable in the environment.

## Introduction

When an organism forages in its environment to obtain food and resources, it cycles between two distinct phases. First, it explores the environment (the exploration phase) and then it uses the collected information to exploit the resources that it has discovered (the exploitation phase). This exploration/exploitation trade off can occur many times in a foraging bout but, in each case, a distinct shift in behavior occurs when the valued resource suddenly becomes readily available. At that point, exploration ceases and the organism focuses on the stimuli or actions that produce the desired outcome.

The medial prefrontal cortex (mPFC) may be a key site to begin a search for neural mechanisms associated with foraging decisions. Rats with anterior cingulate cortex (ACC) lesions decreased the amount of food collected in a competitive or non-competitive food foraging test (Li et al., 2012), while lesions of the rat ACC impaired memory-based foraging on a radial arm maze (Seamans et al., 1995). Lesions in monkeys have also shown that the ACC has an important role in integrating information and sustaining rewarded responses in dynamical foraging tasks (Kennerley et al., 2006). In addition, Kolling et al. (2012) recently demonstrated that, in humans, the ACC appears to contain information about the average value versus cost of foraging in an environment. Cost estimations may in turn be related to the volatility of reward in an environment, which is also represented within the ACC (Behrens et al., 2007; Hayden et al., 2011). While this work has highlighted the role of the ACC in foraging decisions, less attention has been paid to the changes in neural activity associated with the actual act of exploration versus exploitation. Presumably, the shift from exploration to exploitation marks an important psychological boundary to the animal which may be tracked by the mPFC.

Previous work in both humans and non-human primates has provided evidence that neural activity in the ACC may differentiate between these two modes of behavior. Procyk et al. (2000) trained monkeys to touch targets in a sequence and, after several repetitions, the required sequence changed. One group of neurons in the anterior cingulate sulcus was more active during the search period while another group was more active during the repetition or “exploitation” period. In a different task, that also required alternation between exploration and exploitation, ACC neurons produced differential signals to reward at the beginning of search periods versus the end (Quilodran et al., 2008). In fMRI studies performed in humans (Daw et al., 2006), increased activation was observed on exploratory trials compared to exploitative trials in the frontopolar cortex in a 4-arm bandit gambling task. Interestingly, activity in the frontopolar cortex closely tracked their actual behavior, showing differential activity when they adopted an exploratory versus an exploitative strategy. These data suggests that the frontal cortex responds differently during the act of exploration versus exploitation.

It appears that mPFC neurons, including ACC neurons, exhibit consistent action or outcome correlates when the environment remains stable; however, a significant change in the environment or behavior can induce dramatic and widespread changes in activity. Recently, we trained rats on a cued-operant response rule and, after many trials, switched the rule unexpectedly. The rat was required to search alternatives until it arrived at the correct response-based rule that would produce reward. At about the time the rats switched to the new rule, an abrupt and significant shift in the activity state of the recorded ensemble occurred (Durstewitz et al., 2010). On a different task, (Karlsson et al., 2012) found abrupt and coordinated changes in the activity of mPFC cells that occurred at the point when a prior belief was abandoned in favor of exploration of alternative strategies. Although it is becoming increasingly evident that abrupt shifts occur in mPFC neural activity under various circumstances, it is unclear whether such changes also involve a change in total informational content. With regards to foraging, is it the case that exploration and exploitation are fundamentally represented by differing amounts of information? Intuitively, one would expect that neural networks might exhibit higher entropy (involve more neural states) when the animal is exploring versus when it is exploiting a reliable resource because, in the former, many possible contingencies must be considered whereas the later involves a limited focus on a single contingency. The present study compared two behavioral periods, one in which an environment is explored without food, and another when food becomes available. No decisions were required about when to transition between the two modes of behavior, as we were only interested in the activity state changes when the animal was engaged in the actual act of exploration versus exploitation (as defined by food consumption from a reliable source). Ensemble states, transitions and information were characterized during both periods.

Hidden Markov Models (HMM) are well suited to identify the stable and consistent states of a system as well as the transitions between them. In the present study, we used HMM models to identify activity state patterns in ACC ensembles. An additional advantage of HMMs is that they can provide a probabilistic characterization of identified states through time. As a result, it is possible to calculate the entropy of a system based on the relative probabilities of each of the states (Downarowicz, 2011). In the present study, we defined entropy as the number of states co-existing in ACC ensembles with a given probability at a given time. Systems with higher entropy are considered as more complex (Anand and Bianconi, 2009). We found that distinct Markov states were associated with exploration versus exploitation/consumption and that the switch between these states was abrupt. Furthermore, based on measures of entropy and two independent measures of complexity, activity during exploration was more complex than during exploitation/consumption.

## Materials and methods

### Subjects

Five male Long-Evans rats (Charles River Laboratories, Montreal) weighing between 380 and 450 g were used for the *in vivo* electrophysiological recording experiments. Upon arrival, they were given one week to acclimatize to the colony room before training began. They were housed in an inverted 12-h day/12-h night cycle. Once training began, they were food restricted to 90% of their free-feeding weight and were given *ad libitum* access to water. During recovery from surgeries, they were free fed. Upon full recovery, they were reinstated on a food restriction schedule and were retrained on the task. All procedures were conducted in accordance with the Canadian Council of Animal Care and approved by the Animal Care Committee of the University of British Columbia.

### Behavior

Recording and training sessions took place inside a plexi-glass operant chamber with dimensions of 60 cm in length, 36 cm in width, and 40 cm in height. A pedestal mount pellet dispenser (Med Associates) was placed in the middle of the 60 cm panel and raised 45 cm above ground level. The pellet dispenser was controlled by Med Associates (St. Albans, VT) USB control box, connected to a PC workstation and controlled through Med-PC (St. Albans, VT). It dispensed 45 mg sweet dustless precision pellets (BioServ). After a pellet was dropped from the dispenser into the chamber, it bounced on the floor and rolled to its final resting place which was unpredictable; the pellet distribution in the chamber was essentially uniform with a slight bias to rest opposite to the panel that had the pellet dispenser. When the pellet dropped, it made a distinctive sound that typically caused the rat to orient toward the sound and then search and consume the pellet (4.39 ± 0.65 s, average time to consume after the drop). Rats were trained to do this prior to surgery and then retrained after surgery until they were consuming each pellet in less than 5 s.

**Figure 1:**
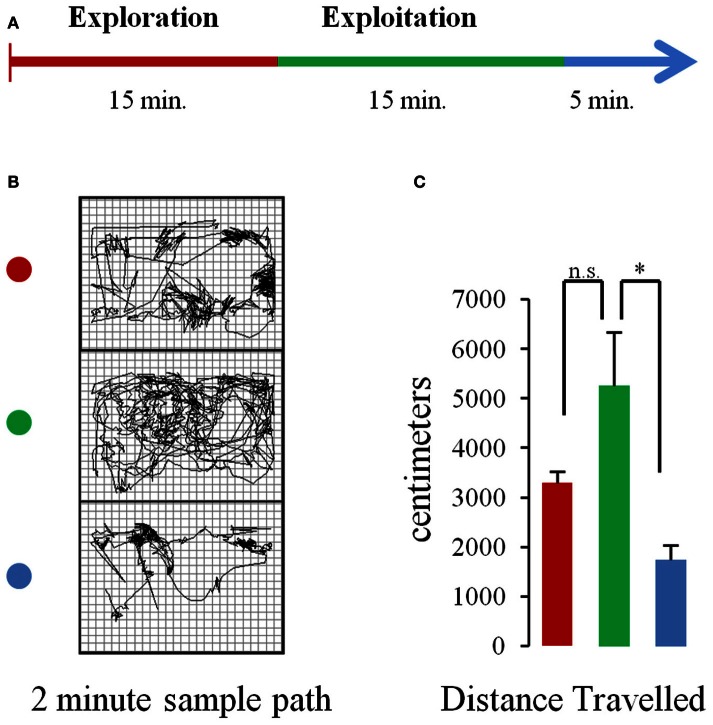
**Task and behavior. (A)** Schematic of the experimental procedure. Rats were allowed to freely explore the chamber for 15 min (baseline period) after which pellets began to fall from a dispenser located above the chamber at a rate of 1 pellet every 10 s (pellet drop period). After 15 min pellet drops ceased. **(B)** Sample paths traveled in 2 min portions of a single session during the baseline (top, red dot), pellet drop period (green dot, middle), and the post-pellet drop period (blue dot, bottom). **(C)** Mean (and s.e.m.) distance traveled for animals across sessions during the first 5 min of the baseline (red bar), pellet drop (green bar), and post-pellet drop period (blue bar). The rats tended to travel more during the pellet drop period than the baseline period although this failed to reach statistical significance. [*p* < 0.05(^*^); not significant (n.s.)].

During recording days, the rat spent 5–10 min in the box, after being tethered and prior to recording, while parameters were adjusted in the Neuralynx system. Recordings started with no overt cue. Rats were allowed to freely explore the operant chamber for 15 min. Then, pellets began to drop from the dispenser above at 10 s intervals for a total of 90 pellets in a 15 minute period. After pellets had been consumed, an additional post-task period of 5 min elapsed where rats could again freely explore their environment before recordings stopped. Figure 1A shows a schematic of the task.

### Surgery and histology

**Figure 2:**
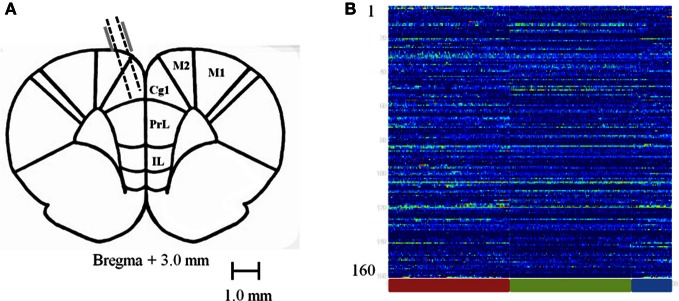
**Recording sites and normalized firing rates. (A)** Diagram of recording sites. Tetrodes were lowered 1.5–2.5 mm at a 15 degree angle into the ACC. **(B)** Carpet plot of 160 recorded neurons pooled across sessions during the baseline (red bar), pellet drop period (green bar), and the post-pellet drop period (blue bar).

Rats were surgically implanted with a custom made 16 tetrode hyperdrive array as previously described in Hyman et al. (2012). Rats were anesthetized under iso-flurane gas, the skull was surgically exposed and a 4 mm by 3 mm hole was drilled and dura removed to expose the brain around coordinates +3.0 mm from Bregma and ±0.5 mm from the midline. The implant was positioned over the area and fixed to the skull with 10 skull screws and dental acrylic. Two additional screws used as ground wires for the implant were placed in the posterior skull. Tetrodes were lowered ~800 μm on the day of surgery; then the rats were given 1–2 weeks of postsurgical recovery time. Tetrodes were then advanced an additional 700–1400 μm to their target location (Figure 2A) and recording sessions took place. After recordings were completed and the experiments had ended, rats were perfused and electrolytic lesions were used to mark the final position of the tetrodes. Brains were then sliced in a cryostat and mounted on slides to confirm anatomical location of tetrode tracts. Exact locations could not be precisely identified because tetrodes were continuously lowered. However, based on tetrode advancement records, the positions were estimated to have been mostly toward the medial wall of the ACC with few crossing into the prelimbic cortex (PL) and none in the medial agranular area (AGm) or infralimbic cortex.

### Electrophysiological recordings

**Figure 3:**
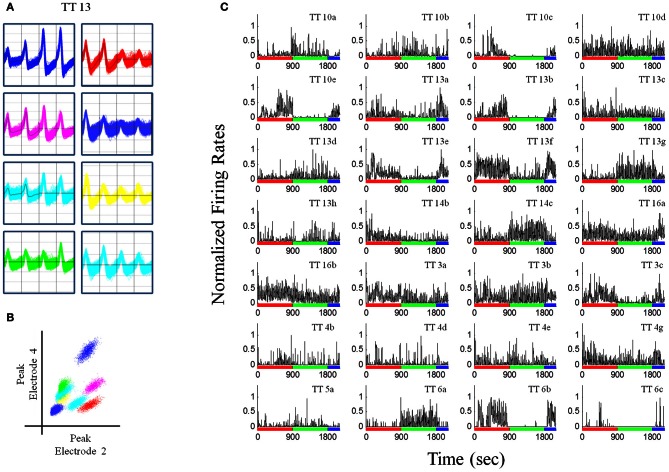
**Recordings from a pellet drop session. (A)** Waveforms recorded from a single tetrode (tetrode #13). **(B)** Plot of peak waveforms from two electrodes of tetrode 13 showing clustering indicative of putative individual units. Tetrode 13 was chosen because it had the largest number of putative individual units. **(C)** Normalized firing rates for unit recordings in one session (*N* = 28), during the baseline (red bar), pellet drop period (green bar), and the post-pellet drop period (blue bar). Note that many units exhibited a robust and almost all-or-none increase (TT14c, TT13g, TT6a) or decrease (TT13e, TT13f, TT10e, TT6b, TT13b) in firing at the point when pellets began to fall at 900s. Bin size = 1 s.

Recordings were obtained using a Neuralynx System. Tetrodes were attached to EIB-36TT boards, plugged into two HS-36 headstages and electrical signals were sent via tether cables to a Digital Lynx 64-channel system and then to a PC workstation. Electrophysiological data and behavioral events were captured using Cheetah 5.0 software. Files were exported into Offline Sorter (Plexon, Inc.) and were manually sorted based on three-dimensional projections of wave form peaks and valleys (Figures 3A,B). Once cells had been sorted, they were exported to Neuroexpler 3.266 (Nex Technologies) and then to Matlab for further analysis.

When rats had recovered from surgery, they were plugged into the system and tetrodes were slowly advanced into the mPFC over an approximately one month period until a stable ensemble was found. Once all tetrodes were placed into the mPFC and were showing stable neural activity, data collection commenced.

### Data analysis

Spike timestamps were converted to instantaneous firing rate (iFR) vectors as described previously in Lapish et al. (2008) and Durstewitz et al. (2010). To obtain an estimate of the neural firing rate for each isolated cell i as a function of time bin *t, ri*(*t*), all spike trains were convolved with Gaussian kernels (*SD* = 500/4 ms) and binned at 500 ms (approximately the inverse of the average firing rate of ≈1.8 Hz). Neurons with average firing rates below 0.1 Hz were excluded from further analysis. A low frequency cut was made for two technical reasons. First, it eliminated cells that were not active during the recording session. Second, when running the HMM scripts, cells that had many “zeros” in the firing rate matrix produced errors. For population analysis, population vectors *r*(*t*) = [*r*1(*t*) … *rN*(*t*)] were formed, with *N* number of single units isolated from a given recording session.

### Hidden markov models

A Markov chain is a dynamic probabilistic system that transitions between Q states through time. The transition from one state to the next is given by a transition probability measure and all possible transitions are contained in the transition matrix. A HMM follows the same properties, with the added component that the Q states are not directly observed and instead must be inferred from the observed variables. The observed variables determine which state the system is in at any given time. In neural data, the observed variable is the firing rate and the hidden states and their underlying dynamics are assumed to correspond to the neural processes that are being computed.

To compute the parameters of the HMM model, we used the HMM Matlab Tool Box provided by Murphy (1998, available on-line). In this toolbox, the transition matrix is calculated using the Baum–Welch Expectation Maximization (EM) algorithm using a random transition matrix and random prior probabilities as a starting point. The emission probabilities for a given state are assumed to be drawn from an N-dimensional normal distribution (for a system of *N* neurons) where both the mean and the covariance matrix are determined during the EM step. We assumed a diagonal covariance matrix for each state. Parameters are updated on each iteration until the log-likelihood converges. At that point, a forward–backward algorithm is applied to the transition matrix in order to calculate the posterior probabilities. The posterior probabilities are given by *P* [*Q*(*t*) = *i*|*y*(1 : *T*)], where *y*(1 : *T*) is the observed data over the entire time period, *T*. It is important to note that the most likely path (calculated by the viterbi algorithm) gives one state as the most likely, while the posterior probabilities give the probability that each state is present at each time point. For further explication of how HMM can be applied to neural or other kinds of data refer to Rabiner (1989) for the classical tutorial and Murphy (2012) for a modern perspective.

### Bayesian information criteria

One hurdle that has limited the applicability of HMMs to neural data is the decision of the number of states the model should use. It is important to find the optimal number of states without over-fitting. Various heuristics have been employed (see Visser et al., 2002) to address this issue. Typically, one would look at the likelihood at all states and choose the state at which it asymptotes. However, more states will typically lead to continuously increasing likelihoods. Two goodness of fit analysis that have been successfully performed on neural data (Xydas et al., 2011) are the Akaike Information Criteria (AIC) and the Bayesian Information Criteria (BIC). In the present study, the BIC was used to find the best trade off. BIC places a cost on the number of parameters in the model (number of states given the number of neurons, number of observations, covariance and elements of transition matrix). The BIC attains either an asymptote or a minimum for the model that best explains the data. We calculated this goodness of fit model by applying the following formula:

$$\begin{array}{l} BIC=-2\times L+log\left(\text{length}\left(iFR\right)\right)\;\times (Q\times (N)\;+\;Q\times \left(N\right)\\ \;+Q\times \left(Q-1\right)) \end{array}$$

Where *Q* is the number of states, *N* is the number of neurons, and *L* is the maximized log-likelihood found from the EM algorithm. The second term represents the number of fit parameters in the model.

### Entropy

Entropy is a measure that was originally formulated for thermodynamic systems, but later was shown to be equivalent to information as defined by Shannon (1948). The information in a system is a measure of how many states it represents in bits or nats and is defined, with the latter units, as:

$$\text{Entropy}=-\sum_{i\;=\;1}^{Q}P_{i}\times lnP_{i}$$

In the above equation, the *P*_*i*_ represents the probability that the system of neurons is in a particular state *i*. We use the HMM model described above to determine the probabilities of the neurons being in a particular state at a given time *t*. These probabilities correspond to the posteriors. The entropy is an indicator of the number of states with non-zero posterior probabilities at any given time; the maximum entropy is achieved when all states are equiprobable, while the minimum entropy is attained when only one state is probable. We smooth the posterior probabilities with a sliding centered average of 3 bins (500 ms each) before calculating entropy and, in order to improve visualization for full time series plots, we smooth at 61 bins (500 ms each).

### Combining iFR sessions

We combined all recorded sessions into one single session by aligning all iFRs to the starting time of the task when the first pellet drops. This leads to a combined iFR trace that consists of an approximately 15 min pre-task period, followed by 15 min of pellet drops and then a post-task period. Combining all recordings led to a system of 160 neurons (Figure 2B). This merged dataset was used in evaluating the Kolmogorov Complexity (KC) and PCA complexity.

### Kolmogorov complexity

An alternative measure to entropy is KC that, in the appropriate limit, indeed converges to the entropy. It measures the size of the minimal computer program needed to represent a piece of data. In other words, it measures how big does a program need to be to generate the observed data—the larger the program the more complex the original data source. Although it is not possible to compute the minimal program directly, it is possible to estimate an upper bound on the KC using standard compression algorithms. In this paper, we use the compression program bzip2, though other compression algorithms have been shown to yield similar results. For each iFR trace, we compute the KC of a sliding time window. For each time window, we output the corresponding iFR trace to a file, compress the file and then output the compressed size of the file in bytes. This size is our estimate of KC at each time point.

### PCA complexity

Principal component analysis (PCA) is a standard technique for doing dimensionality reduction on high-dimensional data sets. Indeed, it forms the basis of several compression algorithms. It is based on diagonalizing the covariance matrix of the dataset, yielding the dominant directions in the high-dimensional space along which the data varies most. Here, we use PCA to calculate the effective number of components that exist within an iFR signal at a given time point as another measure of data complexity/entropy. We performed PCA on the iFR matrix and then for each iFR time bin, determined the loadings on each principal component (PC). We counted the number of PCs needed to capture 90% of the observed signal at each time point; this was done by sorting the loadings in descending order and then summing until the sum was greater than 90% of the total sum. Where the sum starts is a measure of how many components exist in the data at each time. In order to more clearly see how the number of components changes throughout the experimental trial, we apply a simple smoothing procedure to compute a sliding average of components with time.

## Results

The database used for analysis was composed of 160 neurons with a mean firing rate of 1.84 ± 0.95 Hz, recorded from 5 rats over 8 sessions, with an average neural ensemble size of 20 units. During each session, rats were allowed to explore the enclosure without food for 15 min. Thereafter, food pellets fell from a dispenser above at a rate of one/10 s. Each pellet would fall, hit the plexi-glass floor and bounce until it settled at a random location within the enclosure. The rat would then search, retrieve, and consume each pellet. The rats would continue until the 90 pellets that were available to them in a session were depleted (Figure 1A). Hence the task involved exploration of an enclosed chamber followed by exploitation/consumption of a food source. Figure 1B shows the paths a rat took during the baseline, pellet drop and post pellet drop period of a single session. The average distance traveled during the first 5 min of the baseline, pellet drop and post pellet drop period for three rats across four sessions is shown in Figure 1C. Although rats tended to travel further in the pellet-drop than the baseline period, this effect just missed significance [*t*_(3)_ = 3.08, *p* = 0.054].

Rats had experience with the 90 pellet task before the recording sessions commenced. Figure 3 shows neural recordings obtained during a single pellet drop session. There were a multitude of different responses (Figures 2B, 3C), as one would expect from the mPFC; however, there were notable cases of neurons that abruptly turned on or off as soon as the food pellets started dropping from above. This was suggestive of a transition in activity state patterns at the point when food became available.

### Ensembles exhibit discrete states during exploration versus exploitation

**Figure 4:**
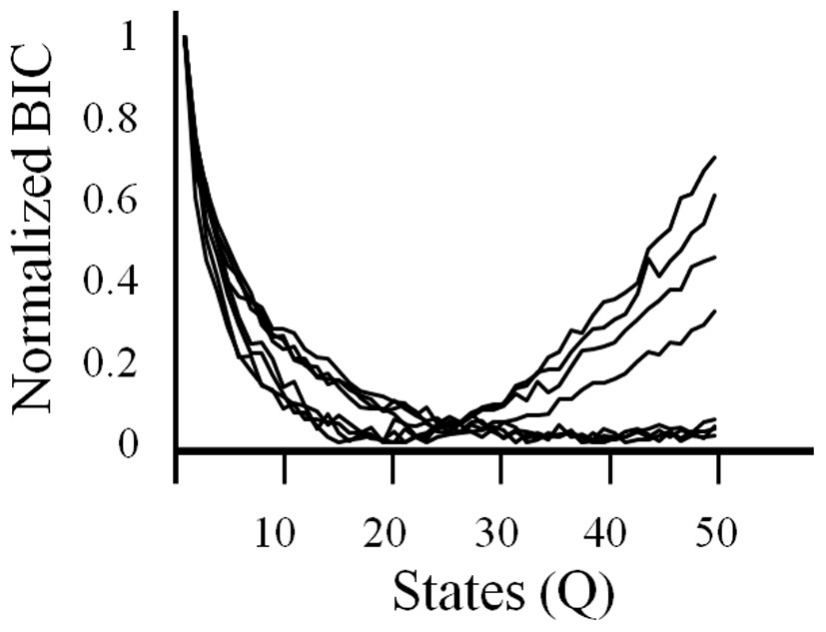
**The criteria used to select the number of states in the HMM.** A Bayesian Information Criteria (BIC) was used to estimate the optimal number of states (Q) to be used in the HMM for each session. BICs are normalized.

In order to get a better understanding of overall ensemble activity states and transitions between them, an HMM analysis was employed. With HMMs, the goodness of fit can be strongly affected by the number of model parameters. Therefore, a criteria is used that imposes a cost on the number of parameters, such as the BIC. Using this criteria, the log likelihood constrained by the BIC was variable across sessions but always showed an asymptote or minima (Figure 4). BIC was normalized in order to facilitate comparison between ensembles. The model with the optimal number of states as given by the asymptote or minima of the BIC was used in the analysis described below.

**Figure 5:**
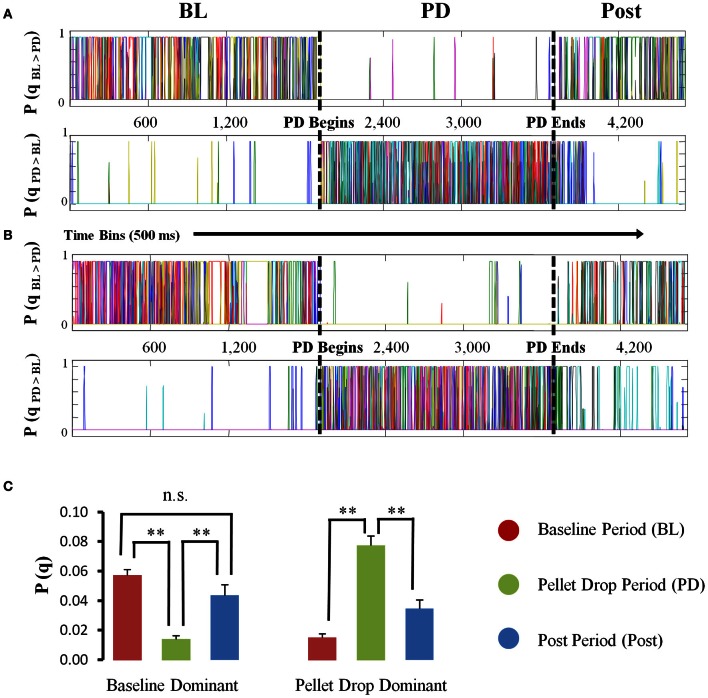
**State probabilities present throughout pellet drop sessions.** (**A** and **B**) Posterior probabilities for two sample sessions. For each session, the states derived from the HMM were divided into one of two groups (“baseline dominant” or “pellet drop period dominant”), based on whether their posterior probabilities were larger (top panel **A,B**) for the baseline period (denoted by a BL) versus the pellet drop period (denoted by a PD) or vice versa (bottom panel **A,B**). **(C)** Group means (and s.e.m.) comparing the posterior probabilities for “baseline dominant” (BL > PD) versus “pellet drop period dominant” (PD > BL) states during the baseline and pellet drop period across all sessions. States either had significantly strong probabilities during the baseline period or the pellet drop period. [*p* < 0.005(^**^); not significant (n.s.)].

The HMM analysis showed that states were usually present either during the baseline period or the pellet drop period (Figure 5A). States were placed into one of two categories, “baseline dominant” or “pellet-drop dominant,” respectively, based on whether or not their average posterior probabilities were larger for the baseline periods than an equivalent interval from the pellet drop period. Virtually all of the states in every session were much more dominant either during the baseline or pellet drop periods. In order to show that these categories were robust across sessions, we did the following: the average posterior probabilities for all states in each category were calculated separately for the baseline period and for the pellet drop period. As shown in Figure 5B, states placed in the “baseline dominant” category had approximately four times larger average posterior probabilities during the baseline period (0.0574 ± 0.0037) than during the pellet drop period (0.0140 ± 0.0023); *t*_(117)_ = 9.13, *p* < 0.005. States that were present in the baseline period were also present in the post period and the magnitude of their probabilities were not significantly different; 0.0574 ± 0.0037 and 0.0437 ± 0.0071 respectively, *t*_(117)_ = 1.88, *p* > 0.05 (Figure 5C). Conversely, states placed in the “pellet drop dominant” category had approximately five times larger average posterior probabilities during the pellet drop period (0.0775 ± 0.0063) than during the baseline period (0.0150 ± 0.0027); *t*_(81)_ = 8.07, *p* < 0.005. An all-or-none collective shift from one group of neural states to another is striking and suggests a fundamental change in the operation of the medial PFC at the point when the animal moves from exploration to exploitation of a food source.

### Entropy and complexity is lower during consumption/exploitation than exploration

**Figure 6:**
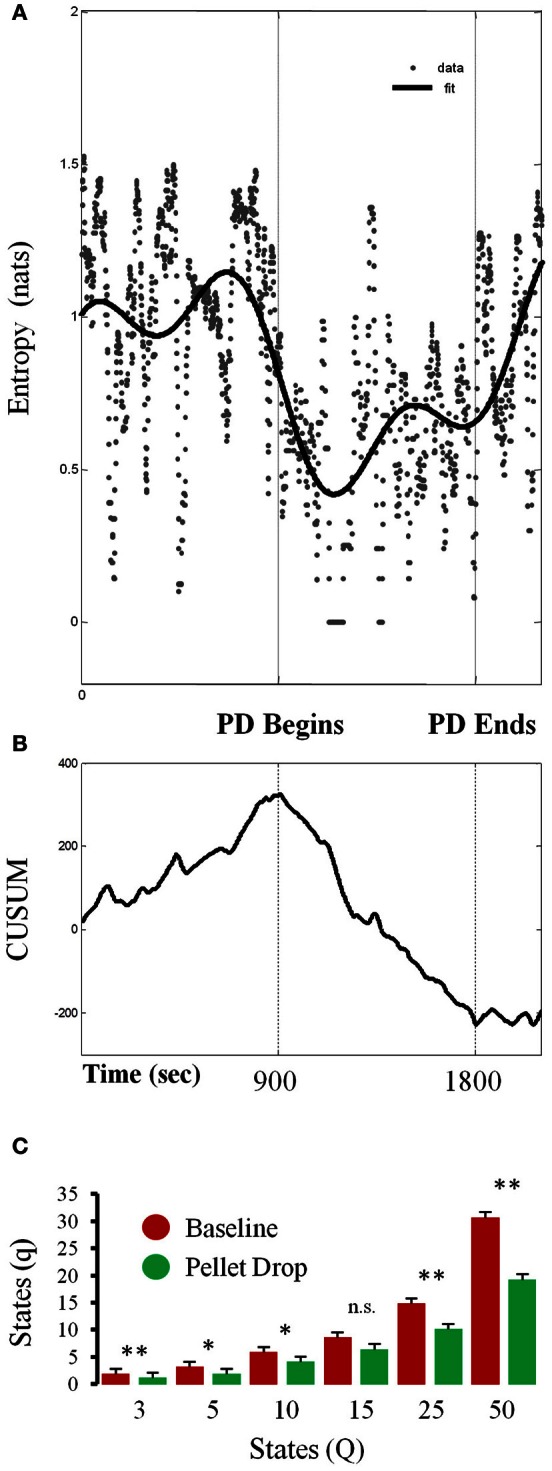
**Changes in entropy throughout pellet drop sessions. (A)** Entropy calculated from the posterior probabilities derived from a 7 state HMM for a single session. **(B)** Time series analysis indicating the moment at which entropy shifts, which corresponds to the moment of pellet drop onset. **(C)** Data from all sessions showing the average number of states with non-zero posterior probabilities (q) during the baseline period (red bars) and pellet drop period (green bars) as derived from HMMs with differing numbers of states (Q). The number of states was usually smaller for the pellet drop period than the baseline period. [*p* < 0.05(^*^); *p* < 0.005(^**^); not significant (n.s.)].

As shown in Figure 6A, entropy calculated for a single session from a 7 state model abruptly decreased at the point when the pellets started to fall from above. In order to detect the moment at which this transition occurred, a time series analysis was performed (Figure 6B), with the cumulative sum showing a peak at the time the pellet begins to drop. In this session, the system transitioned through more states during exploration relative to consumption/exploitation. In terms of group statistics, one problem with calculating entropy based on Markov state probabilities across sessions is that such calculations are quite sensitive to the number of states in the model. In order to control for this, entropy was calculated for each session using different numbers of HMM states. As shown in Figure 6C, entropy was usually significantly lower during exploitation than exploration for models with different numbers of states.

**Figure 7:**
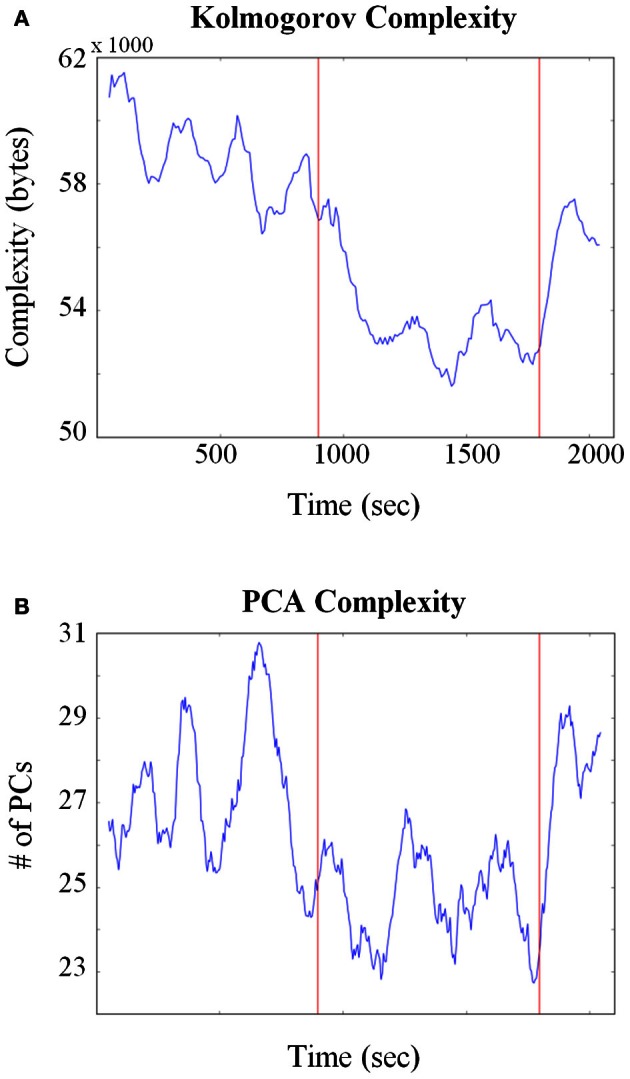
**Changes in neural complexity throughout pellet drop sessions. (A)** All neurons from all sessions were pooled and Kolmogorov complexity (KC) was calculated over time using a sliding window of size = 150 s. The KC decreased at the point the pellets began to fall (first red line) and increased when they stopped falling (second red line). **(B)** A principle component analysis (PCA) was calculated on all neurons pooled from all sessions. The average number of principle components (PCs) needed to reconstruct 90% of the iFR decreased as soon as pellets started to fall (first red line) and increased again after they stopped falling (second red line).

Given the potential issues associated with calculating entropy from HMM states, other approaches were also employed. A related quantity to entropy is KC that measures the size of the minimal program or procedure needed to generate the observed pattern or activity (Kolmogorov, 1968). One advantage of KC is that it does not require an explicit evaluation of the probabilities of the states that exist within the system. It has found a variety of applications in biology such as to compare complexities between different genomic sequences (Li et al., 2001; Vinga and Almeida, 2003; Penner et al., 2011) and molecular structure comparisons (Krasnogor and Pelta, 2004). Figure 7A plots the KC for all neurons combined across all sessions (Total units = 160) and shows that at the point the pellets started dropping, neural complexity decreased. In order to test for significance between behavioral epochs, a Kolmogorov–Smirnov (KS) test was applied between corresponding complexity time series. The exploitation/consumption period was significantly lower (*p* ≈ 1e-33) than the baseline period. However, the post consumption period was also significantly lower than the baseline period (p ≈ 1e-13).

A principle component analysis (PCA) based approach was also employed to quantify neural complexity for the combined 160 neuron group. PCA was performed on the iFR matrix and the number of PCs that were needed to reconstruct 90% of the firing rate were calculated through time. As shown in Figure 7B, fewer PCs were required to reconstruct the firing rate activity when the rat transitioned from exploration to exploitation. A KS test (*p* ≈ 1e-20) verified that the exploitation period was indeed lower than the baseline period. No significant difference was found between baseline and post pellet drop period. Therefore, both entropy and PCA-based analysis showed that the baseline and post periods were similar. Even though the KS analysis of KC showed that the post period was different than the baseline, it should be emphasized that the post period was still significantly higher than the exploitation/consumption period.

It is important to note that the decrease in neural entropy or complexity measured using these approaches was not due to a decrease in overall movement, since distances traveled actually were greater during the exploitation phase when the rats searched for pellets in the chamber than during the exploration phase (Figure 1C).

### Local changes in neural entropy for the period surrounding each pellet drop

**Figure 8:**
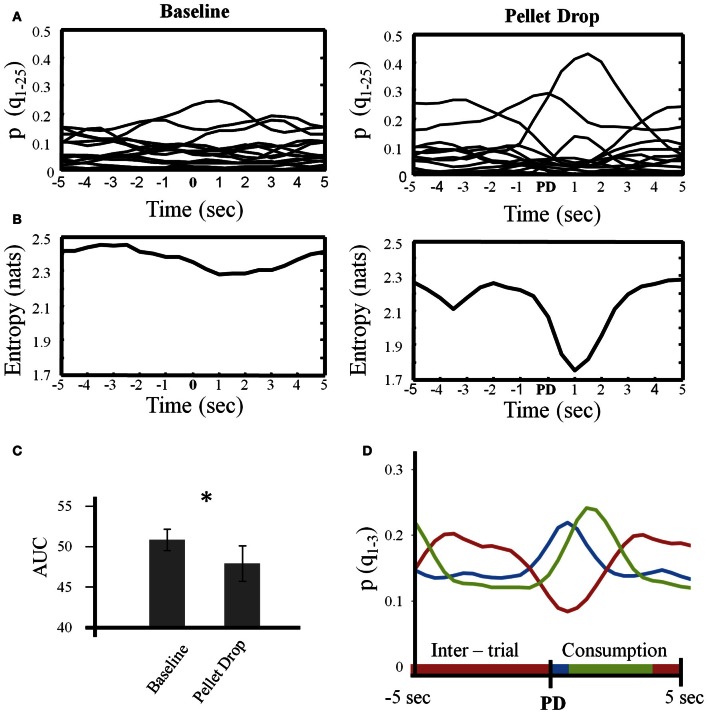
**State dynamics in the 10 s period surrounding each pellet drop. (A)** For a single session, the posterior probabilities of all states (*Q* = 25) were individually averaged across repeated 10 s intervals during the baseline period (left) and during repeated 10 s intervals centered on the pellet drop during the pellet drop period (right). **(B)** Entropy calculated based on the average posterior probabilities shown in **(A)**. Note that local entropy decreased just prior to the pellet drop and reached a minimum just after it. **(C)** Group data from all sessions showing that the area under the entropy curves was significantly smaller for the peri-pellet drop period than for an equal number of 10 s intervals during the baseline period. **(D)** The three largest probability states for the peri-pellet drop period were extracted from each session and averaged. In every session, there was a consistent state that decreased during the peri-pellet drop interval (red line), a state that peaked just around the time a pellet hit the floor (blue line), and a state that peaked somewhat later around the time when the rats consumed the pellet (green). ^*^p <0.05.

The analysis above provided a description of the neural state changes across entire sessions. During the pellet drop period, the important event was of course the pellet drop and its imminent consumption. Figure 8 focuses on the state dynamics surrounding each pellet drop. As shown in Figure 8A for a single session, multiple low probability states were present during the baseline exploration period while a few states tended to emerge more strongly during different parts of the peri-pellet drop interval. When entropy was calculated in this interval, there was an abrupt and significant drop at the point when the pellet hit the floor (Figure 8B). In order to quantify the drop in entropy across sessions, the area under the entropy curves (AUC) was calculated for repeated 10 s intervals during the baseline period and for 10 s periods surrounding each pellet drop. The average AUC for repeated 10 s intervals from the baseline period was significantly higher at 49.98 ± 1.21 compared to 47.25 ± 2.04 for the 10 s intervals surrounding the pellet drops [*t*_(7)_ = 2.63, *p* < 0.05], (Figure 8C).

**Figure 9:**
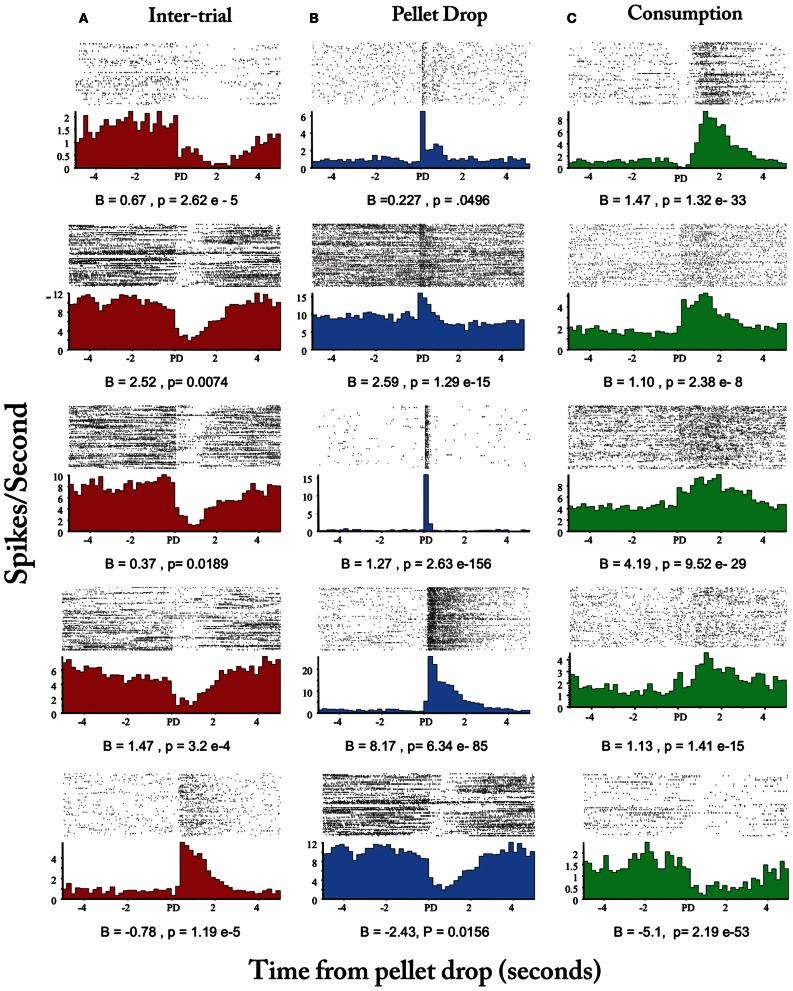
**Peri-event histograms of individual neurons selective for intertrial interval, pellet drop, and consumption states.** Neurons selected based on beta coefficient significance are shown in **(A)** for the inter-trial interval factor (red), **(B)**, for pellet drop factor (blue), and **(C)** for the consumption factor (green). Top four rows correspond to neurons with positive beta coefficients while the last row corresponds to neurons with negative ones.

Finally, Figure 8D plots the means of the three states with the largest posterior probabilities in the peri-pellet drop intervals across all sessions. One state was relatively flat with a peak roughly aligned to the time when the pellet hit the floor; a second state emerged strongly when the pellet was found and consumed while a third state was less likely around the actual time of the pellet drop, but emerged during the inter-pellet drop interval. Since this is group data from all 8 sessions, these states were highly reproducible and consistent. Thus, the decrease in entropy during exploitation occurred because of the emergence of a few clear and highly probable exploitation-related states in all sessions. To get an idea of how individual neurons mapped onto the three states shown in Figure 8, a linear regression with three factors corresponding to intertrial, pellet drop and consumption periods was applied to individual iFR vectors. Example neurons from different sessions that attained the highest significance (as determined by *p*-values of the *t*-statistic) are plotted in Figure 9. The top 4 rows correspond to cells that had positive coefficients (B) while the bottom row represents cells with negative ones (–B).

## Discussion

The present study investigated the ensemble activity state patterns in the mPFC as rats were exploring an open chamber or exploiting a reliable food source. A HMM was used to parse ensemble activity into discrete states over time. Distinct and largely independent states emerged when the rat was in an environment that contained food versus when it did not. Overall, entropy or complexity decreased as rats went from exploring the environment to exploiting the reliable food source. This decrease occurred largely because of the emergence of strong and stable activity states that were associated with key motivation events.

### The strengths and limitations of HMMs applied to neural data

HMMs have been previously used to detect stable and recurrent states in neural networks. They are well suited for this application because neural activity is thought to evolve through a sequence of states. The underlying assumption is that the states detected by HMMs are functionally meaningful in that they are each associated with the representation of a particular type of information. As a result, if one can detect the sequence of states and characterize how they transition through time, it reveals something about the local neural code. With this in mind, Radons et al. (1994) used HMMs to characterize neural activity from the visual cortex and were able to predict with high levels of accuracy which visual stimuli were being presented to an anesthetized monkey. Abeles et al. (1995) applied HMMs to a working memory task and in so doing identified several quasi stationary states in monkey frontal cortex that corresponded to behavioral and sensorial events. Subsequently, Seidemann et al. (1996) showed that the unique states detected by an HMM were specific to a monkey's response in a delayed localization task and that they could be used to predict the monkey's response correctly on ~90% of trials. HMMs have also been applied to elucidate the functioning of other brain areas; Jones et al. (2007) showed that rodent gustatory cortical ensembles progress through a sequence of stimulus-specific sequences and Camproux et al. (1996) used it to classify bursting activity of locus coeruleus neurons under different pharmacological conditions. Finally, another interesting approach of HMMs can be found in Kemere et al. (2008), where they were used to interpret neural activity in the motor cortex related to planning and movement to enable control of external devices by the brain. This last finding raises the possibility that HMMs can be used, not only to acquire theoretical knowledge of brain processes, but also to translate it into practical terms in order to improve the quality of life of patients with spinal cord injury or neurodegenerative diseases.

Yet in spite of the potential of HMMs, they have not been widely used as a means to quantify cortical activity. One reason is likely because of the difficultly in knowing how many states should be used in the model (Rainer and Miller, 2000). Previous studies of frontal activity have used models with less than 10 states successfully (Abeles et al., 1995; Seidemann et al., 1996; Rainer and Miller, 2000; Xydas et al., 2011). The present study used the BIC that places a cost on excessive model parameters. While in some data sets, <10 states appeared optimal, >20 states were optimal in others. A larger number of states may have been required here because previous studies were performed in well-trained chair-restrained monkeys whereas in the present study, rats were free to move around the enclosure. Such free flowing, poorly constrained naturalistic behavior may necessitate the monitoring of many more information sources and hence involve more states detectable with the HMM.

HMM-based entropy calculations are also strongly dependent on the number of model states. While HMMs with different numbers of states tended to show that the transition to exploitation was associated with a drop in entropy, this was not always the case (Figure 5B). This could have been because activity may not have been optimally parsed in different sessions. Therefore, two completely independent means of calculating neural complexity were used. One was based on PCA where the number of PCs required to 90% of neural firing rate was calculated through time. The second, termed KC, was borrowed from computer science and can be estimated using compression algorithms commonly used to compress computer files. The idea is that a less complex file can be compressed to a smaller size than a more complex file. In the present case, the algorithm was able to compress neural iFR data to a smaller size during exploitation than during exploration. Hence the three independent measures converged on a similar result.

### Implications of changes in neural complexity during exploration and exploitation

In spite of its shortcomings, the calculation of entropy based on the posterior probabilities of the HMM is perhaps most intuitive of the three measures. Posterior probabilities give an estimation of the likelihood that a given state is present at a given time. If the posterior probability is 1 for a given state, it would be 0 for all of the other states and in this case entropy across all states at that time would be 0. Alternatively, if 2 or more states have non-zero posterior probabilities, then entropy would increase. A decrease in entropy during consumption/exploitation implies that fewer states are estimated as likely during this period. This can be appreciated by examining Figure 8A where many states, each with low posterior probabilities, were present during the baseline period, but fewer states emerged with larger posterior probabilities in the peri-pellet drop period. The states emerging in the peri-pellet drop period had clear associations with behaviorally significant events such as the pellet drop, pellet consumption and the inter-pellet drop interval. Thus, the present results suggest that in mPFC ensembles, exploration is associated with many weak representations while consumption/exploitation is characterized by a few strong representations.

The conclusion is conceptually similar to the predicted effects of dopamine on network activity in the PFC (Durstewitz et al., 2000; Durstewitz and Seamans, 2004, 2008). Specifically, our “dual state” model predicted that low levels of dopamine alter activity such that multiple weak representations co-exist in PFC nearly simultaneously while moderate elevations in dopamine levels decrease the number of representations present at any one time, but those that remain would be strongly enhanced. Although this theory was developed as a way to understand PFC activity on working memory tasks, the computational models on which it was based were derived from the known biophysical properties of cortical circuits and their modulation by dopamine and therefore are equally applicable to any situation where PFC dopamine levels vary. With relevance to the present study, moderate elevations in PFC dopamine levels occur at the time when food is introduced (Feenstra and Botterblom, 1996; Taber and Fibiger, 1997; St. Onge et al., 2012) and in this sense, the present results are in good agreement with model predictions.

### The abrupt nature of coherent transitions in mPFC ensembles

One important realization that came out of the early applications of HMMs to frontal cortex activity was that activity states could switch very abruptly (Abeles et al., 1995; Seidemann et al., 1996). The present study provided another demonstration of this phenomenon. The detection of abrupt transitions was not an artifact of HMMs, however, similar types of abrupt transitions have been detected in the mPFC using a number of different techniques. Using a foraging-based decision-making task on a radial arm maze, we observed that statistically unique and distinct activity states emerged during different task epochs (Lapish et al., 2008). For instance, a unique activity state pattern consistently emerged at arm decision points yet abruptly transitioned to a different pattern within ~1 s when the rat reached the food cup at the end of the arms. A widespread and abrupt transition in mPFC activity state patterns was also observed to occur near the point when rats seized upon a new operant action rule after a trial and error search period (Durstewitz et al., 2010) as well as when a prior belief was abandoned in favor of exploration of alternative strategies (Karlsson et al., 2012). Abrupt and coordinated activity state transitions were also observed when rats switched from one unique context to another (Hyman et al., 2012). The magnitude of the shift observed in the present study was larger than the shifts associated with different task rules or task epochs, but of comparable magnitude to those associated with changing contexts. The appearance of food for a hungry rat can reasonably be assumed to reflect a fairly substantial event and perhaps the magnitude of the shift may be related to the significance of the associated behavioral event.

### The role of the mPFC in foraging

In humans, the ACC appears to contain information about the average value versus cost of foraging in an environment (Kolling et al., 2012). This is consistent with the neural correlates observed in the primate ACC as ACC neurons might weigh the relative value of leaving a depleting resource for a new one (Hayden et al., 2011). ACC neurons were found to reach a fixed threshold at the time when it is optimal for patch-leaving. In addition, it has been previously shown that exploration/exploitation modes of behavior can recruit differential forms of neural activity and ensembles (Procyk et al., 2000; Averbeck et al., 2006; Daw et al., 2006; Brown et al., 2007; Quilodran et al., 2008; Kolling et al., 2012). In this paper, we present similar results. However, one fundamental difference is the lack of a decision component. In terms of optimal foraging theory, there is a trade-off between exploration and exploitation while in the present study, there is really no trade off because, even if the animal decided to stop eating and explore, there is no cost associated with that decision. The value of the approach applied in this paper lies in the attempt to characterize information. We not only show that there are different neural states for exploratory and exploitative/consumptive behaviors, but also that a differential information load exists. The next step would be to apply the methods employed in this paper to a trial an error task and quantify how information changes as cost/benefit evaluations take place.

The findings in this paper provide insight into how the ACC may inform foraging decisions. While foraging decisions are related to a number of variables such as cost and value, these variables are in turn determined by the prevailing internal state. Food cues only have value if they signal food and if the animal is hungry. The present study speaks more to the relevance of the ACC to the monitoring of internal states than to the neural mechanisms involved in deciding when to explore or exploit. Specifically, it was the appearance of food to a hungry rat that appeared to evoke a widespread and coherent shift in ACC activity states. Such shifts likely change the processing mode of ACC networks in a broad manner. Aside from the food itself, the same sensory information was available to the rat before and after pellets started dropping, yet their presence profoundly changed the way that information was represented in the ACC. If food is unlikely or uncertain, multiple stimuli or actions must be considered. This may be reflected as an increase in entropy or complexity. Conversely, if a food source suddenly becomes reliable, most of these candidate stimuli or actions can be ignored and all neural resources can be directed to those that are reliably related to reward. This appears to be reflected as a decrease in entropy or complexity. Thus, widespread changes in activity states in the mPFC may help to imbue otherwise arbitrary stimuli or actions with increased prominence or value.

### Conflict of interest statement

The authors declare that the research was conducted in the absence of any commercial or financial relationships that could be construed as a potential conflict of interest.
